# Topical application of RTA 408 lotion activates Nrf2 in human skin and is well-tolerated by healthy human volunteers

**DOI:** 10.1186/s12895-015-0029-7

**Published:** 2015-07-14

**Authors:** Scott A. Reisman, Angela R. Goldsberry, Chun-Yue I. Lee, Megan L. O’Grady, Joel W. Proksch, Keith W. Ward, Colin J. Meyer

**Affiliations:** Reata Pharmaceuticals, Inc., 2801 Gateway Dr. Ste 150, Irving, TX 75063 USA

**Keywords:** RTA 408 lotion, Nrf2, Radiation dermatitis, Cancer supportive care

## Abstract

**Background:**

Topical application of the synthetic triterpenoid RTA 408 to rodents elicits a potent dermal cytoprotective phenotype through activation of the transcription factor Nrf2. Therefore, studies were conducted to investigate if such cytoprotective properties translate to human dermal cells, and a topical lotion formulation was developed and evaluated clinically.

**Methods:**

*In vitro*, RTA 408 (3–1000 nM) was incubated with primary human keratinocytes for 16 h. *Ex vivo*, RTA 408 (0.03, 0.3, or 3 %) was applied to healthy human skin explants twice daily for 3 days. A Phase 1 healthy volunteer clinical study with RTA 408 Lotion (NCT02029716) consisted of 3 sequential parts. In Part A, RTA 408 Lotion (0.5 %, 1 %, and 3 %) and lotion vehicle were applied to individual 4-cm^2^ sites twice daily for 14 days. In Parts B and C, separate groups of subjects had 3 % RTA 408 Lotion applied twice daily to a 100-cm^2^ site for 14 days or a 500-cm^2^ site for 28 days.

**Results:**

RTA 408 was well-tolerated in both *in vitro* and *ex vivo* settings up to the highest concentrations tested. Further, RTA 408 significantly and dose-dependently induced a variety of Nrf2 target genes. Clinically, RTA 408 Lotion was also well-tolerated up to the highest concentration, largest surface area, and longest duration tested. Moreover, significant increases in expression of the prototypical Nrf2 target gene NQO1 were observed in skin biopsies, suggesting robust activation of the pharmacological target.

**Conclusions:**

Overall, these data suggest RTA 408 Lotion is well-tolerated, activates Nrf2 in human skin, and appears suitable for continued clinical development.

**Electronic supplementary material:**

The online version of this article (doi:10.1186/s12895-015-0029-7) contains supplementary material, which is available to authorized users.

## Background

Nuclear factor erythroid 2-related factor 2 (Nrf2) is the principal transcription factor that regulates the expression of greater than 90 % of all antioxidative genes [1]. Activation of Nrf2 induces the expression of a battery of such genes, which results in a coordinated intrinsic cellular defense effort to switch to a phenotype that protects against oxidative and electrophilic insult, highlighted by increased antioxidative capacity, induction of glutathione (GSH) synthesis, increased energy production, and elimination of potentially harmful molecules [2, 3]. Activation of Nrf2 also imparts potent anti-inflammatory properties in cells through detoxification of reactive oxygen species (ROS), which activate the pro-inflammatory transcription factor nuclear factor-kappa B (NF-κB) [4].

Semi-synthetic oleanane triterpenoids are among the most potent activators of Nrf2 identified to date; they bind to specific cysteine residues on Kelch-like ECH-associated protein 1 (Keap1), resulting in subsequent translocation of Nrf2 to the nucleus, where it binds to specific antioxidant response elements, facilitating induction of a multitude of cytoprotective genes [5–8]. Recently, a newly developed semi-synthetic oleanane triterpenoid denoted RTA 408 has been described as an activator of Nrf2 in rat skin [9]. Specifically, topical application of RTA 408 to rats at concentrations of 0.1, 1, or 3 % led to significant and dose-dependent induction of many Nrf2 target genes in the skin, including the prototypical Nrf2 targets NAD(P)H:quinone oxidoreductase (Nqo1), sulfiredoxin 1 (Srxn1), and the rate-limiting enzyme subunits for the synthesis of GSH, namely glutamate-cysteine ligase, catalytic and modifier subunits (Gclc and Gclm, respectively). Further, immunohistochemical methods demonstrated that increased staining for Nqo1 protein and total GSH of structures in both the epidermis and dermis was consistent with full transdermal penetration of RTA 408 [9].

The ability to activate Nrf2 and produce cytoprotection was also observed in mice in a model of fractionated radiation-induced dermatitis [10]. Topical application of RTA 408 was highly effective at decreasing the severity of dermatitis in mice exposed to fractionated radiation, where doses as low as 0.01 % RTA 408 demonstrated significant improvements, and a dose of 1 % RTA 408 restored normal appearance of skin by the end of the 40-day treatment period, including substantial hair regrowth [10]. The remarkable improvement in skin attributable to topical treatment with RTA 408 was associated with significant increases in antioxidative Nrf2 target mRNA expression in skin. In contrast, the same skin samples had marked decreases in pro-inflammatory NF-κB target mRNA expression. Overall, these data support the development of RTA 408 as a new therapy for the prevention and treatment of radiation-induced dermatitis in cancer patients undergoing radiation therapy and other conditions associated with oxidative stress and inflammation.

Nevertheless, the translatability of the effects of RTA 408 to human cells has yet to be evaluated. Therefore, this study was performed to investigate the effects of RTA 408 on activation of Nrf2 after exposure of human primary keratinocytes *in vitro* and cultured human skin explants *ex vivo*. Positive results from these *in vitro* and *ex vivo* studies in human cells and tissues suggested the efficacy of topical RTA 408 observed in the mouse model of fractionated radiation-induced dermatitis could be translated to humans. Therefore, a clinically feasible lotion formulation of RTA 408 was developed for a Phase 1 clinical study to evaluate safety, pharmacokinetics (PK), and pharmacodynamics (PD) after topical application to healthy volunteers.

## Methods

### Culturing of primary human keratinocytes

Fresh human abdominal skin, obtained from a Caucasian donor undergoing an abdominoplasty (female aged 46 years old) was collected and maintained in a holding medium consisting of HEPES buffered DMEM containing antibiotics and antifungals. The epidermis was removed from the dermis after overnight incubation at 4 °C with dispase in the holding medium and then underwent trypsin digestion. The keratinocyte suspension was seeded on 3 T3 fibroblasts and rendered mitotically inactive by mitomycin C treatment. Human keratinocyte monolayers were cultured for 6 days in DMEM (2 mM Ca^2+^), with 10 % FCS, L-glutamine (2 mM), insulin (5 μg/mL), hydrocortisone (0.4 μg/mL), epidermal growth factor (EGF, 10 ng/mL), penicillin (100 IU/mL), and streptomycin (100 μg/mL), at 37 °C, in 95 % air/5 % CO_2_ atmosphere, with 95 % relative humidity. After 6 days, the 3T3 feeder cells were removed from the human keratinocyte cultures by a trypsin/EDTA treatment. The next day, the human keratinocytes were removed by trypsinization. The human keratinocyte suspension was centrifuged, resuspended in fresh culture medium, and then strained through a series of filters. Cells (1.5x10^5^/well) were then seeded into 96-well plates with 200 μL of EpiLife® medium and 60 μM calcium, antibiotics, and human keratinocyte growth supplement (HKGS) per well. Human tissue samples for the isolation of keratinocytes (described above) and skin explants (described below) were obtained in accordance with the Human Tissue Act of 2004 (England Wales and Northern Ireland) and collected with the appropriate donor consent.

### Treatment and harvesting of primary human keratinocytes

RTA 408 (3, 30, 100, 300, 700, and 1000 nM), vehicle (DMSO, 0.1 % final concentration), or nothing (media control) was incubated with cells in two separate 96-well plates (1.5 x 10^5^ cells per well) for 16 h. At the time of harvest, one plate utilized the MTT assay (Invitrogen, V13154) to examine cell viability, and one plate was processed for QuantiGene Plex 2.0 analysis (*i.e.*, mRNA expression of Nrf2 target genes), according to manufacturer’s instructions and as previously reported [9, 11].

### Culturing of human skin explants

Skin, obtained from a female reduction mammoplasty (48 year old donor), was collected and placed in a holding medium consisting of HEPES buffered DMEM containing antibiotics and antifungals. The fat was removed, and a 5-mm punch biopsy was used to cut 30 biopsies. Biopsies were cultured at the air-liquid interface with 5 mL of DMEM media (2 mM Ca^2+^), 10 % FCS, L-glutamine (2 mM), insulin (5 μg/mL), hydrocortisone (0.4 μg/mL), EGF (10 ng/mL), penicillin (100 IU/mL), and streptomycin (100 μg/mL), at 37 °C, in a 95 % air/5 % CO_2_ atmosphere, with 95 % relative humidity. The dermis of each culture was immersed in the media, while the epidermis was in contact with air.

### Ex vivo treatment and harvesting of human skin explants

Skin cultures were split into 5 treatment groups. RTA 408 (0.03, 0.3, or 3 %), vehicle (sesame oil), or nothing (media control) was applied topically twice daily for 2 days and once on Day 3. Approximately 50 μL of RTA 408 or vehicle was applied to the entire surface of the skin cultures. Prior to each application, a visual inspection of the skin cultures confirmed there was no residual RTA 408 or vehicle from the previous administration. All skin cultures were harvested 8 h after the final administration on Day 3, with half of the replicates fixed for 24 h in phosphate-buffered formalin (pH 7.0–7.4), transferred to 70 % ethanol, then processed and paraffin-embedded, according to standard histological techniques. The remaining skin samples were snapped frozen.

### Quantigene 2.0 Plex mRNA expression analysis

Messenger RNA (mRNA) was quantified using Quantigene Plex 2.0 technology according to manufacturer’s protocol (Affymetrix, Inc., Santa Clara, CA) and as previously described [12]. Probe sets were designed against the human genome for analysis of Nrf2 target genes, and a modified version of Panel 11834 (Affymetrix) was used. Human primary keratinocyte data were normalized to the housekeeping gene PPIB. Human skin explant data were normalized to the average of housekeeping genes RPL13A and PPIB.

### Immunohistochemical analysis of NQO1 protein in cultured human skin explants and biopsies

Levels of NQO1 protein in skin sections were determined by immunohistochemistry (IHC) using previously described methods [9]. NQO1 staining intensity of 5X magnification photomicrographs was quantified using ImageJ software v1.46 with the Densitometry 1 plug-in, both freely available from the National Institutes of Health (http://rsbweb.nih.gov/ij/index.html).

### Healthy volunteer clinical study design

The clinical study (https://clinicaltrials.gov/ct2/show/NCT02029716) enrolled healthy adults (male and female) aged 18 to 65 years, with Fitzpatrick skin type I to IV, and a body mass index (BMI) between 18 and 32 kg/m^2^. Demographic data are presented in Table 1. The study was conducted sequentially in 3 parts to assess the safety, tolerability, PD, and PK of RTA 408 Lotion applied topically twice daily (BID), at 8:00 a.m. and 8:00 p.m., for up to 28 days. For each application, the appropriate amount of lotion was applied to the skin and gently massaged for the appropriate time (Parts A and B: 10–15 s; Part C: 45 s). The areas of application were allowed to dry for 5 min, and then the entire area was covered with loose-fitting gauze to keep the lotion confined during normal activities.

**Table 1:** Phase 1 Clinical trial healthy volunteer baseline characteristics

Demographic/characteristic category/statistic	Part A	Part B	Part C	Total
N	12	10	10	32
Age				
Mean	41.9	40.2	42.0	41.4
S.D.	10.1	9.7	8.4	9.2
Median	38.5	40.5	40.0	39.0
Minimum	29	23	33	23
Maximum	59	53	59	59
Gender (N)				
Female	1 (8.3 %)	1 (10.0 %)	2 (20.0 %)	4 (12.5 %)
Male	11 (91.7 %)	9 (90.0 %)	8 (80.0)	28 (87.5 %)
Ethnicity				
Hispanic or Latino	1 (8.3 %)	0 (0.0 %)	2 (20.0 %)	3 (9.4 %)
Not Hispanic or Latino	11 (91.7 %)	10 (100.0 %)	8 (80.0 %)	29 (90.6 %)
Race				
White	11 (91.7 %)	10 (100.0 %)	8 (80.0 %)	29 (90.6 %)
Asian	1 (8.3 %)	0 (0.0 %)	0 (0.0 %)	1 (3.1 %)
Other	0 (0.0 %)	0 (0.0 %)	2 (20.0 %)	2 (6.3 %)
Fitzpatrick Skin Type				
I (0–7)	0 (0.0 %)	1 (10.0 %)	0 (0.0 %)	1 (3.1 %)
II (8–16)	4 (33.3 %)	5 (50.0 %)	4 (40.0 %)	13 (40.6 %)
III (17–25)	8 (66.7 %)	4 (40.0 %)	6 (60.0 %)	18 (56.3 %)
IV (26–30)	0 (0.0 %)	0 (0.0 %)	0 (0.0 %)	0 (0.0 %)
Baseline BMI				
Mean	27.9	27.7	25.9	27.2
S.D.	2.7	3.2	3.2	3.1
Median	27.6	28.5	27.3	27.7
Minimum	22.6	21.8	21.0	21.0
Maximum	31.2	31.3	29.8	31.3

Part A was a randomized, double-blind, placebo-controlled assessment of the safety, local skin tolerability, PD, and PK of 3 concentrations of RTA 408 Lotion (0.5 %, 1 %, and 3 %, w/w) compared to lotion vehicle (0 %) applied topically to 12 healthy subjects BID for 14 days to a small skin surface area on the lower back (4 cm^2^ for each concentration). Part B was open-label and assessed the safety, tolerability, PD, and PK of the highest tolerated dose of RTA 408 Lotion from Part A (*i.e.*, 3 %) applied topically to 10 healthy subjects BID for 14 days to a larger skin surface area on the lower back (100 cm^2^). During the dosing period in Parts A and B, subjects were confined to the study site for 15 days. Part C was open-label and assessed the safety, tolerability, PD, and PK of RTA 408 Lotion (3 %) concentration applied BID to an even larger skin surface area (500 cm^2^) on the backs of 10 healthy subjects for 28 days. During the dosing period in Part C, subjects were confined to the study site for 29 days. Total daily doses of RTA 408 for Parts A, B, and C were approximately 1.8, 30, and 150 mg/day, respectively. The assessment of safety was based primarily on the incidence, intensity, and type of adverse events, Modified Draize Skin Irritation Assessments, clinical laboratory assessments (hematology, clinical chemistry, and urinalysis), physical examinations, 12-lead electrocardiograms (ECG), and vital signs.

### Analysis of RTA 408 plasma concentrations in healthy volunteers following topical administration of RTA 408 lotion

Blood samples for PK analysis were collected for determination of plasma RTA 408 concentrations prior to dosing and 1, 2, 4, 12, and 24 h after the first topical application of RTA 408 Lotion on Days 1, 7, and 14 of Parts A, B, and C and also on Day 28 for Part C. A validated LC/MS/MS method with a lower limit of quantitation (LLOQ) of 0.074 ng/mL and an upper limit of quantitation (ULOQ) of 37.0 ng/mL was used for quantification of RTA 408 in plasma samples.

### Analysis of NQO1 protein expression in healthy volunteer skin punch biopsies

Punch biopsies (3 mm) for evaluation of induction of NQO1 protein expression, the prototypical Nrf2 target gene, were collected the day following the final dose in each part of the study. A local injection of lidocaine HCl (1 %) was used for anesthesia. Biopsies were incubated in formalin at room temperature for 24 h and then transferred to 70 % ethanol. NQO1 IHC on the skin biopsies was performed as described above.

The protocol and informed consent documents were submitted to and approved by the duly constituted Western Institutional Review Board prior to initiation of the clinical study. The study was conducted in accordance with the Declaration of Helsinki and with all applicable laws and regulations of the locale and country where the study was conducted, and in compliance with Good Clinical Practice Guidelines.

### Statistics

Nrf2 target gene data were analyzed with Sigmaplot 12.0 (Systat, Inc., San Jose, CA) by student’s *t*-test or by one way-analysis of variance (ANOVA) followed by Duncan’s Multiple Range post-hoc test with significance set at *p* < 0.05.

## Results

### RTA 408 induces Nrf2 target genes in primary human keratinocytes

**Fig. 1:**
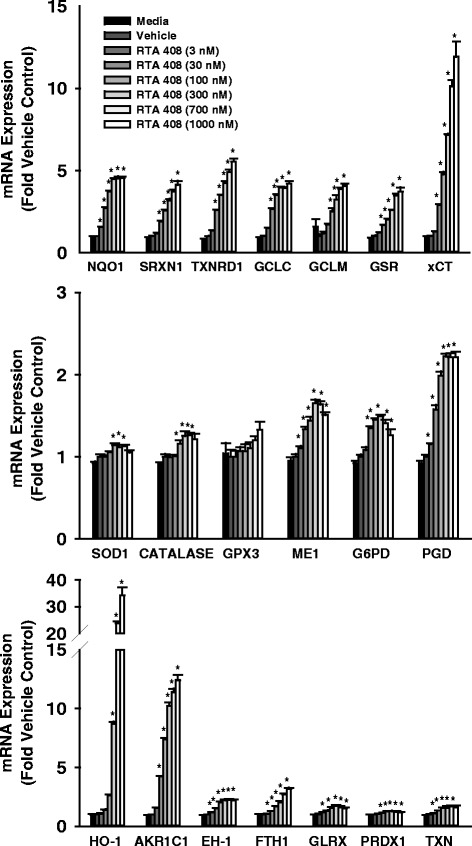
Effect of RTA 408 on mRNA Expression of Nrf2 Target Genes in Primary Human Keratinocytes. Freshly isolated primary human keratinocytes were incubated with RTA 408 (3–1000 nM) or vehicle (DMSO, 0.1 % v/v) for 16 h and analyzed for mRNA expression of Nrf2 target genes. Data were normalized to the housekeeping gene PPIB and are presented as mean fold vehicle control ± standard error of the mean (S.E.M.). *p < 0.05 vs. vehicle control

The effects of RTA 408 (3–1000 nM) on the mRNA expression of Nrf2 target genes were evaluated in freshly isolated primary human keratinocytes. There were no differences in percent cell viability among the groups (Additional file 1: Figure S1), indicating that RTA 408 was well-tolerated over the concentration range tested. Further, RTA 408 significantly induced the mRNA expression of many cytoprotective Nrf2 target genes in a concentration-dependent manner (Fig. 1). Remarkably, for most of the genes evaluated [*i.e.*, NQO1, SRXN1, thioredoxin reductase (TXNRD1), GCLC, GCLM, glutathione reductase (GSR), xCT, heme oxygenase-1 (HO-1), aldo-keto reductase 1C1 (AKR1C1), and ferritin heavy chain 1 (FTH1)], significant induction was observed beginning towards the lower range of the concentrations tested (*i.e.*, 3 and/or 30 nM), and induction continued to increase dose-dependently up to the highest concentration tested (*i.e.*, 1000 nM). Though the levels of induction of superoxide dismutase 1 (SOD1), catalase, glutathione peroxidase 3 (GPX3), epoxide hydrolase-1 (EH-1), glutaredoxin (GLRX), peroxiredoxin 1 (PRDX1), and thioredoxin (TXN) are less than other Nrf2 target genes, it is still quite meaningful for this particular subset of antioxidant proteins. Further, the combined coordinated upregulation of the mRNA expression of these antioxidant enzymes would likely have a profound antioxidant and cytoprotective effect on the cells.

### Topical application of RTA 408 induces Nrf2 target genes in human skin explants

**Fig. 2:**
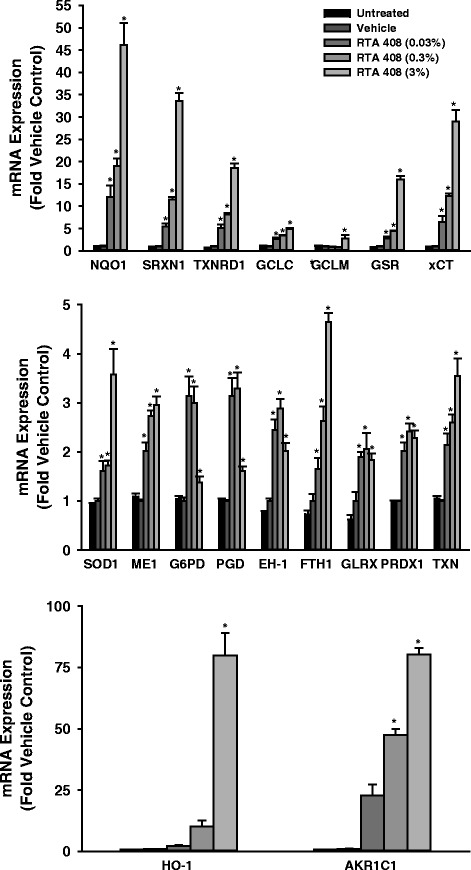
Effect of RTA 408 on the mRNA Expression of Nrf2 Target Genes in Human Skin Explants. Human skin explants from a healthy donor were cultured. RTA 408 (0.03, 0.3, or 3 %) or vehicle (sesame oil) was applied topically up to twice daily for 3 days and skin was then processed and analyzed for mRNA expression of Nrf2 target genes. Data were normalized to the average of housekeeping genes RPL13A and PPIB and are presented as mean fold vehicle control ± S.E.M. *p < 0.05 vs. vehicle control

**Fig. 3:**
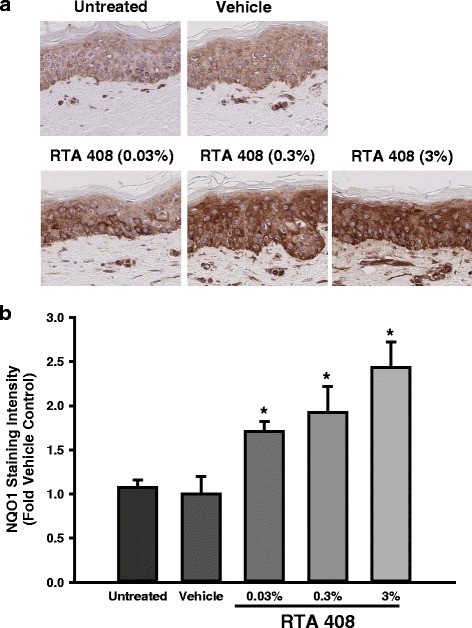
Effect of RTA 408 on the Protein Expression of NQO1 in Human Skin Explants. Human skin explants from a healthy donor were cultured. RTA 408 (0.03, 0.3, or 3 %) or vehicle (sesame oil) was applied topically up to twice daily for 3 days and the skin was fixed in formalin. NQO1 protein was evaluated using standard immunohistochemical techniques. **a**. Representative photomicrographs (20X) are presented for each treatment group. **b**. Staining intensity was determined and presented as mean fold vehicle control ± S.E.M. *p < 0.05 vs. vehicle control

The effects of topical application of RTA 408 (0.03, 0.3, or 3.0 %) were evaluated *ex vivo* in cultured human skin explants. RTA 408 was well-tolerated with skin maintaining normal appearance throughout the treatment period. Eight hours after the last dose, skin was harvested for determination of mRNA expression and NQO1 protein expression by IHC. Similar to the results in primary human keratinocytes, RTA 408 significantly and dose-dependently induced the mRNA expression of a broad panel of Nrf2 target genes (Fig. 2). Very marked (>30-fold) induction of Nrf2 target genes such as NQO1, SRXN1, xCT, HO-1, and AKR1C1 was observed. The mRNA induction of the prototypical Nrf2 target gene NQO1 translated to significant and dose-dependent induction of NQO1 protein in the epidermis of the skin explants (Fig. 3). Statistically significant increases in most Nrf2 target genes at both the mRNA and protein levels were observed at the lowest concentration tested (*i.e.*, 0.03 %).

### Topical application of RTA 408 lotion was well-tolerated and produced low systemic exposures in healthy volunteers

A Phase 1 study was completed to evaluate the safety, local PD, and systemic PK of RTA 408 following topical application of RTA 408 Lotion to healthy volunteers (*n* = 32). RTA 408 Lotion was well-tolerated, with only 1 (3.1 %) subject exhibiting mild application site erythema and pruritus (Table 2 and Additional file 2: Table S1). This subject was enrolled in Part C of the study, receiving 3 % RTA 408 Lotion twice daily for 28 days. This subject had a modified Draize Score of 1 (barely perceptible, faint to pink) on Days 5–9, which subsided on Day 10 with continued RTA 408 Lotion administration, and was not observed during the remainder of the study with continued dosing through Day 28. Modified Draize Scores for all other subjects on all other days were 0 (no erythema). There were no severe adverse effects, and no subjects discontinued treatment of RTA 408 Lotion. With the exception of one subject, all individual abnormal laboratory results were considered to not be clinically significant. In Part C, one subject experienced an adverse event (not related to study drug) of increased alanine transaminase (ALT) levels (79 IU/L) on Day 7. As a result, study drug was stopped on Day 16; study drug was not restarted, but the subject completed the study. The subject also had elevated ALT (48 IU/L) on Day −1, which was recorded in the subject’s medical history. The subject’s ALT value at the end of the study (58 IU/L) was similar to the baseline value (48 IU/L), and the adverse event of increased alanine transaminase (ALT) levels was considered resolved and unrelated to study drug. Further, at no time during the study did this subject have measureable concentrations (<LLOQ of 0.074 ng/mL) of RTA 408 in plasma. Moreover, there were no clinically significant mean changes in vital signs or electrocardiogram (ECG) parameters (heart rate and PR, RR, QRS, QT, QTc, and QTcF intervals) from baseline in any subject, and there were also no clinically significant drug-related abnormalities in clinical laboratory parameters. Overall, RTA 408 was well-tolerated at the doses and durations tested.

**Table 2:** Overview of adverse events

Category	Part A	Part B	Part C	Total
Preferred Term	(*N* = 12)	(*N* = 10)	(*N* = 10)	(*N* = 32)
	N (%)	N (%)	N (%)	N (%)
Subjects with Any Adverse Event	1 (8.3)	3 (30.0)	7 (70.0)	11 (34.4)
Subjects with Any Study Drug Related Adverse Event	0 (0.0)	0 (0.0)	1 (10.0)	1 (3.1)^a^
Subjects with Any Serious Adverse Event	0 (0.0)	0 (0.0)	0 (0.0)	0 (0.0)
Subjects with Any Study Drug Related Serious Adverse Event	0 (0.0)	0 (0.0)	0 (0.0)	0 (0.0)
Subjects with Any Adverse Event Leading to Discontinuation	0 (0.0)	0 (0.0)	0 (0.0)	0 (0.0)

^a^In total, 1 (3.1 %) subject, in Part C, had an RTA 408 Lotion-related adverse event, described as mild application site erythema and pruritus (Table 2). This subject had a modified Draize Score of 1 (barely perceptible, faint to pink) on Days 5–9, which subsided on Day 10, and was not observed during the remainder of the study. Scores for all other subjects on all other days were 0 (no erythema)

Topical administration of RTA 408 Lotion produced very low systemic exposures to RTA 408. The highest dose evaluated in the Phase 1 study was 3 % RTA 408 Lotion (or ~150 mg RTA 408) applied to a 500-cm^2^ skin area (~2.6 % of total body surface area (BSA) based on the average total BSA of ~1.92 m^2^ of volunteers in Part C) twice daily for 28 days, which produced plasma concentrations of RTA 408 near or below the LLOQ (0.074 ng/mL) for all healthy volunteers in the study, indicating that topical application of RTA 408 Lotion did not produce any meaningful systemic exposures. Only one healthy volunteer demonstrated measurable plasma RTA 408 concentrations; this subject was enrolled in the Part C cohort that received the highest dose evaluated, and had measurable plasma RTA 408 concentrations only on Day 28. The maximal plasma RTA 408 concentration quantifiable in this healthy volunteer was 0.0943 ng/mL, and the AUC_(0-24h)_ was 0.0019 h*μg/mL. Overall, RTA 408 produced very low systemic exposures.

### Topical application of RTA 408 lotion to healthy volunteers induced NQO1 protein in skin

**Fig. 4:**
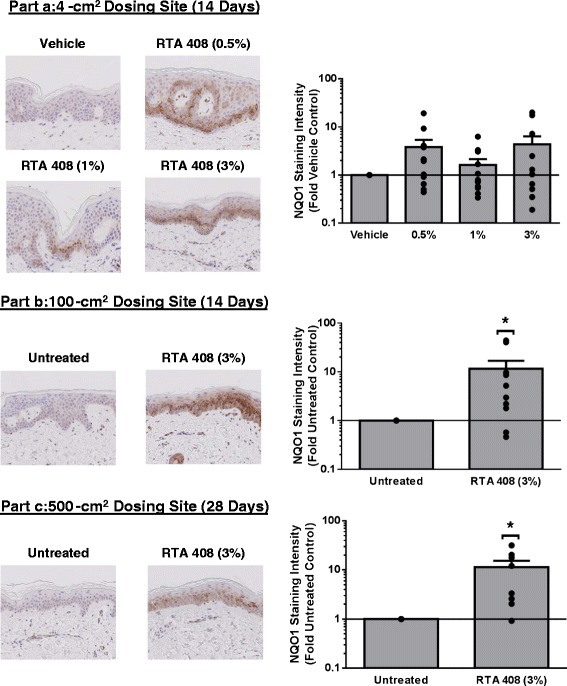
Effect of RTA 408 Lotion on the Protein Expression of NQO1 in Human Skin Biopsies from Phase 1 Clinical Trial. Skin biopsies were collected from healthy volunteers one day after the final dose in Parts **a**, **b**, and **c** of the Phase 1 clinical trial. Part **a** evaluated 3 concentrations of RTA 408 Lotion (0.5, 1, and 3 %) compared to lotion vehicle applied topically to 12 healthy subjects BID for 14 days to a small skin surface area on the lower back (4 cm^2^ for each concentration). Part **b** was open-label and assessed the RTA 408 Lotion (3 %) applied topically to 10 healthy subjects BID for 14 days to skin on the lower back (100 cm^2^). Part **c** was open-label and assessed the RTA 408 Lotion (3 %) applied topically to 10 healthy subjects BID for 28 days to skin on the lower back (500 cm^2^). Representative photomicrographs (20X) from each part are presented on the left with corresponding quantified staining intensities presented on the right. Dots represent individual data points for each subject. Bars present data as mean fold vehicle or untreated control ± S.E.M. *p < 0.05 vs. vehicle or untreated control

Protein expression of NQO1, the prototypical Nrf2 target gene, was evaluated by IHC in skin biopsies collected from all subjects enrolled in each part of the study (Fig. 4). In Part A, RTA 408 Lotion tended to induce the protein expression of NQO1, but high variability precluded statistical significance. In Part A, the relatively small size of the treatment area (4-cm^2^) may have contributed to the variability in NQO1 staining. However, statistically significant induction of NQO1 was observed in Parts B and C when RTA 408 Lotion was applied to larger surface areas (*i.e.*, 100-cm^2^ for Part B and 500-cm^2^ for Part C).

## Discussion

Similar to other semi-synthetic oleanane triterpenoids, RTA 408 is a potent activator of the cytoprotective and antioxidative transcription factor Nrf2 and a potent inhibitor of the pro-inflammatory transcription factor NF-κB [7]. Previous studies have demonstrated that topical dermal application of RTA 408 to rodents produces desirable dermal cytoprotective effects in both the naïve setting in rats and in a mouse model of dermatological injury produced by fractionated radiation exposure [10, 9]. Thus, the present series of nonclinical and clinical studies were conducted to evaluate the translatability of the cytoprotective effects in skin produced by RTA 408 to humans.

Because of their anatomical location within the epidermis, keratinocytes are constantly exposed to external stresses, including sunlight, radiation, and oxygen in air, all of which can contribute to production of excess ROS and ultimately, tissue injury [13]. Thus, human keratinocytes are an important cell type to investigate tolerability of RTA 408 and whether RTA 408 can elicit suitable pharmacologic activation of the Nrf2-mediated antioxidant response. Indeed, freshly isolated primary human keratinocytes demonstrated tolerability (*i.e.*, lack of cytotoxicity) to RTA 408 (3–1000 nM), and RTA 408 produced dose-dependent induction of Nrf2 target genes over the entire concentration range. In addition, these *in vitro* keratinocyte data were consistent with a previous study demonstrating induction of NQO1 protein expression in keratinocytes after topical dermal application of RTA 408 to rat skin [9], suggesting that effects of RTA 408 previously observed in rodent skin may also be observed in human skin.

Full thickness human skin explants more closely mimic the *in vivo* setting and represent a practical model of intact skin that can be used to evaluate topical dermal application to human tissue *ex vivo* [14]. RTA 408 was well tolerated in human skin explants, and topical application of RTA 408 dose-dependently and significantly induced the mRNA expression of Nrf2 target genes. Together, these data demonstrate that topical application of RTA 408 is well tolerated in a relevant non-clinical human skin model and produces robust Nrf2 activation.

Consistent with the nonclinical data, RTA 408 Lotion was very well tolerated, when applied topically to healthy human volunteers up to concentrations of 3 % to a 500-cm^2^ area twice daily for 28 days. Notably, no systemic exposure was generally observed, suggesting that the pharmacological effects of RTA 408 were limited to locally treated skin sites. Finally, protein induction in skin biopsies of the prototypical Nrf2 target gene NQO1 was associated with administration of RTA 408 Lotion, indicating that local Nrf2 activation can be achieved in human skin.

The profound Nrf2 activation effects of RTA 408 in skin are consistent with, although more potent than, the activity previously observed with the polyphenol phytochemical and weak Nrf2 activator curcumin. Curcumin also activates Nrf2 and induces Nrf2 target genes when incubated with human primary keratinocytes or cultured skin biopsies, though less potently and efficaciously than RTA 408 (15). Curcumin was tested clinically in a recently completed trial investigating the effects of oral administration (6 g/day) to breast cancer patients undergoing radiation therapy (16). This high dose of curcumin only modestly reduced radiation dermatitis severity and moist desquamation, but overall, the data suggested that pharmacological activation of Nrf2 in skin may be a beneficial strategy for the prevention and treatment of radiation dermatitis (16). Therefore, a more potent activator of Nrf2, such as RTA 408, may provide the necessary level of cytoprotection to provide a meaningful clinical benefit.

Based in part on these results, RTA 408 Lotion has been advanced into clinical evaluation for the prevention and treatment of radiation dermatitis in cancer patients receiving radiotherapy (https://clinicaltrials.gov/ct2/show/NCT02142959). In such a radioprotection setting, one theoretical concern could be that such robust induction of the cytoprotective response may afford protection to cancer cells, as well as normal skin cells. However, available nonclinical data suggest that this will not be the case. A recent study evaluated the radioprotective effects of RTA 402, a potent Nrf2 activator and closely related analog to RTA 408, in normal epithelial cells and a panel of cancer cells exposed to ionizing radiation [15]. RTA 402 evoked significant Nrf2-dependent radioprotection in normal lung and breast epithelial cells, as well as lymphocytes, but provided no protection nor activated Nrf2 in any of the cancer cells evaluated. This suggests that RTA 402 and RTA 408 differentially affect Nrf2 in normal versus cancer cells. Similarly, RTA 408 increases survival and protects the rat gastrointestinal tract from a lethal dose of whole body irradiation [16], while also inhibiting growth of established xenografts with enhanced anti-cancer effects when coupled with radiation treatments [16]. Overall, these data suggest that RTA 408 will afford radioprotection to normal cells only and may enhance radiosensitivity to cancer cells; additional work to characterize these differential activities is ongoing.

## Conclusions

Collectively, the present data demonstrate that topical application of RTA 408 Lotion is well-tolerated by healthy human volunteers, and RTA 408 Lotion produces an appropriate pharmacodynamic response of Nrf2 activation in skin that would be hypothesized to be cytoprotective under conditions of oxidative stress and inflammation. These safety data, coupled with the profound pharmacology observed in a clinically-relevant rodent model of radiation-induced skin injury [10], support the continued clinical development of topical RTA 408 Lotion.

## Additional files

Additional file 1: Figure S1. After a 16 h incubation with RTA 408 (3–1000 nM), human primary keratinocytes were analyzed using a 3-(4,5-Dimethylthiazol-2-yl)-2,5-diphenyltetrazolium bromide (MTT) assay to examine cell viability. Data are presented as mean % vehicle control (n=5/group) ± S.E.M. No differences in viability were observed among groups. Additional file 2: Table S1. Adverse events in phase 1 clinical trial in healthy volunteers.

## Abbreviations

AKR1C1: Aldo-keto reductase 1C1
ALT: Alanine transaminase
ECG: Electrocardiogram
EH-1: Epoxide hydrolase-1
FTH1: Ferritin heavy chain 1
G6PD: Glucose-6-phosphate dehydrogenase
GCLC: Glutamate cysteine ligase, catalytic subunit
GCLM: Glutamate cysteine ligase, modifier subunit
GPX3: Glutathione peroxidase 3
GSH: Glutathione
GSR: Glutathione reductase
HO-1: Heme oxygenase-1
IHC: Immunohistochemistry
Keap1: Kelch-like ECH-associated protein 1
LLOQ: Lower limit of quantitation
ME1: Malic enzyme 1
NF-κB: Nuclear factor-kappa B
NQO1: NAD(P)H: quinone oxidoreductase 1
Nrf2: Nuclear factor erythroid 2-related factor 2
PD: Pharmacodynamics
PGD: 6-phosophoglucose dehydrogenase
PK: Pharmacokinetics
PRDX1: Peroxiredoxin
ROS: Reactive oxygen species
SOD1: Superoxide dismutase 1
SRXN1: Sulfiredoxin
TXN: Thioredoxin
TXNRD1: Thioredoxin reductase 1
ULOQ: Upper limit of quantitation
xCT: Cystine/glutamate transporter
