# Supplementary figures and images for: Topical application of RTA 408 lotion activates Nrf2 in human skin and is well-tolerated by healthy human volunteers
Source: BMC Dermatol. 2015 Jul 14;15:10. doi: 10.1186/s12895-015-0029-7 (PMC4501113; doi:10.1186/s12895-015-0029-7)

Additional file 1: Figure S1

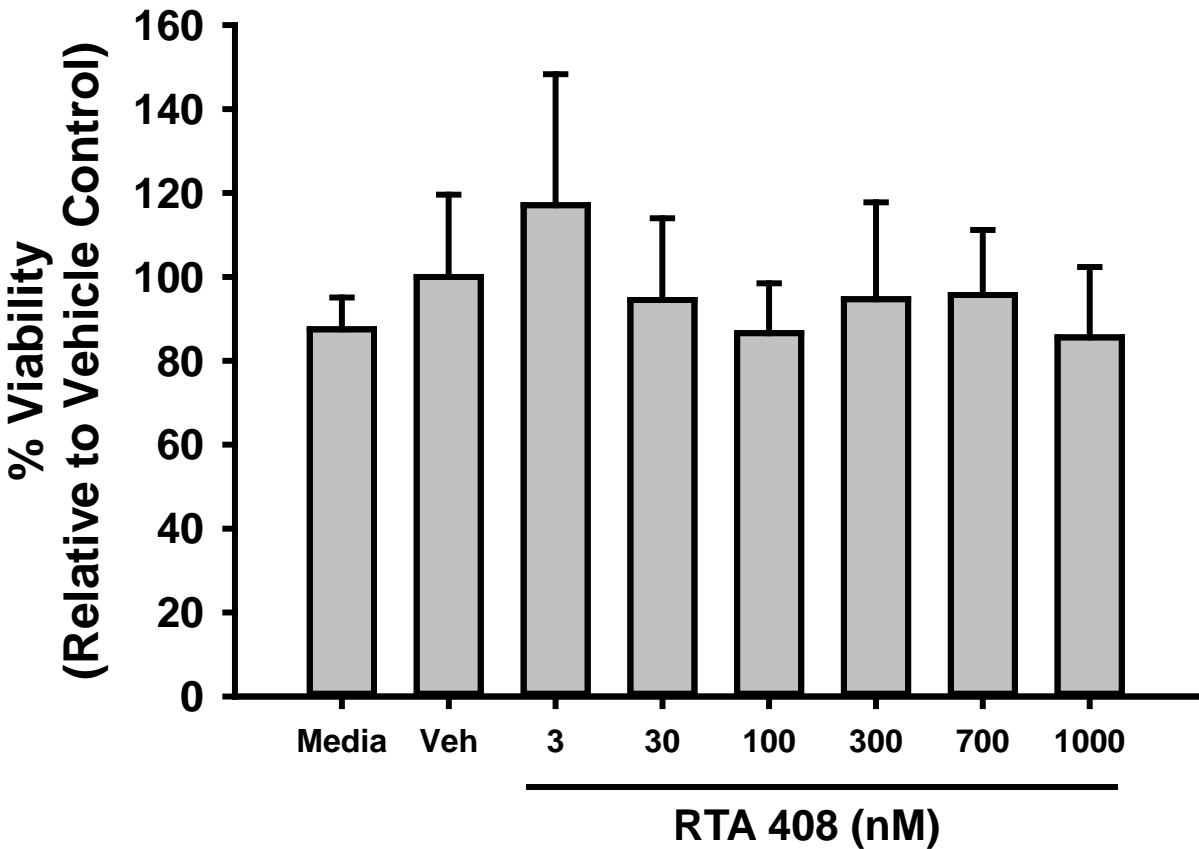

Supplement: Additional file 1: Figure S1. — After a 16 h incubation with RTA 408 (3–1000 nM), human primary keratinocytes were analyzed using a 3-(4,5-Dimethylthiazol-2-yl)-2,5-diphenyltetrazolium bromide (MTT) assay to examine cell viability. Data are presented as mean % vehicle control (n=5/group) ± S.E.M. No differences in viability were observed among groups. [file 12895_2015_29_MOESM1_ESM.pdf]
