# Supplementary material for: Topical application of RTA 408 lotion activates Nrf2 in human skin and is well-tolerated by healthy human volunteers
Source: BMC Dermatol. 2015 Jul 14;15:10. doi: 10.1186/s12895-015-0029-7 (PMC4501113; doi:10.1186/s12895-015-0029-7)
Supplement: Additional file 2: Table S1. — Adverse events in phase 1 clinical trial in healthy volunteers. [file 12895_2015_29_MOESM2_ESM.docx]

**Additional file 2: Table S1 Adverse Events in Phase 1 Clinical Trial in Healthy Volunteers**

| **System Organ Class**  **Preferred Term** | **Part A**  **(N=12)**  **N (%)** | **Part B**  **(N=10)**  **N (%)** | **Part C**  **(N=10)**  **N (%)** | **Total**  **(N=32)**  **N (%)** |
| --- | --- | --- | --- | --- |
| Gastrointestinal Disorders | 0 (0.0) | 0 (0.0) | 1 (10.0) | 1 (3.1) |
| Abdominal Pain | 0 (0.0) | 0 (0.0) | 1 (10.0) | 1 (3.1) |
| General Disorders | 0 (0.0) | 0 (0.0) | 1 (10.0) | 1 (3.1) |
| Application Site Erythema | 0 (0.0) | 0 (0.0) | 1 (10.0) | 1 (3.1) |
| Application Site Pruritus | 0 (0.0) | 0 (0.0) | 1 (10.0) | 1 (3.1) |
| Infections and Infestations | 1 (8.3) | 0 (0.0) | 0 (0.0) | 1 (3.1) |
| Upper Respiratory Tracts Infection | 1 (8.3) | 0 (0.0) | 0 (0.0) | 1 (3.1) |
| Injury, Poisoning, and Procedural Complications | 0 (0.0) | 2 (20.0) | 1 (10.0) | 3 (9.4) |
| Foreign Body | 0 (0.0) | 1 (10.0) | 0 (0.0) | 1 (3.1) |
| Post-Procedural Discomfort | 0 (0.0) | 1 (10.0) | 0 (0.0) | 1 (3.1) |
| Procedural Pain | 0 (0.0) | 0 (0.0) | 1 (10.0) | 1 (3.1) |
| Investigations | 0 (0.0) | 0 (0.0) | 1 (10.0) | 1 (3.1) |
| ALT Increased^a^ | 0 (0.0) | 0 (0.0) | 1 (10.0) | 1 (3.1) |
| Musculoskeletal and Connective Tissue Disorders | 0 (0.0) | 0 (0.0) | 2 (20.0) | 2 (6.3) |
| Neck Pain | 0 (0.0) | 0 (0.0) | 1 (10.0) | 1 (3.1) |
| Pain in Extremity | 0 (0.0) | 0 (0.0) | 1 (10.0) | 1 (3.1) |
| Nervous System Disorders | 1 (8.3) | 1 (10.0) | 3 (30.0) | 5 (15.6) |
| Headache | 0 (0.0) | 1 (10.0) | 2 (20.0) | 3 (9.4) |
| Cervicobrachial Syndrome | 0 (0.0) | 0 (0.0) | 1 (10.0) | 1 (3.1) |
| Dizziness | 1 (8.3) | 0 (0.0) | 0 (0.0) | 1 (3.1) |
| Parasthesia | 0 (0.0) | 0 (0.0) | 1 (10.0) | 1 (3.1) |
| Respiratory, Thoracic, and Mediastinal Disorders | 0 (0.0) | 0 (0.0) | 1 (10.0) | 1 (3.1) |
| Oropharyngeal Pain | 0 (0.0) | 0 (0.0) | 1 (10.0) | 1 (3.1) |
| Skin and Subcutaneous Tissue Disorders | 0 (0.0) | 0 (0.0) | 1 (10.0) | 1 (3.1) |
| Dermatitis Contact | 0 (0.0) | 0 (0.0) | 1 (10.0) | 1 (3.1) |

^a^One subject in Part C was withdrawn from study drug on Day 16 due to an adverse event of increased serum alanine transaminase (ALT) levels; study drug was not restarted but the subject completed the study. The adverse event was not considered related to study drug because ALT levels in this subject were also elevated at Day -1.
